# Keeping adults physically active after Falls Management Exercise (FaME) programmes end: development of a physical activity maintenance intervention

**DOI:** 10.1186/s40814-021-00844-w

**Published:** 2021-05-15

**Authors:** Sarah Audsley, Denise Kendrick, Pip Logan, Elizabeth Orton

**Affiliations:** 1 Division of Primary Care, University of Nottingham, Nottingham, NG7 2RD UK; 2 Division of Rehabilitation, Ageing and Wellbeing, University of Nottingham, Nottingham, NG7 2RD UK

**Keywords:** Older adults, Physical activity, Falls prevention, Intervention development

## Abstract

**Background:**

Falls prevention exercise programmes help to improve muscle strength, balance and physical function, and reduce falling rates in older adults. Improvements in muscle strength, balance and physical function are reversed if older adults do not continue to be physically active after falls prevention exercise programmes end. This paper describes the design process of an intervention that aimed to maintain physical activity in older adults exiting falls prevention exercise programmes.

**Methods:**

The development of the Keeping Adults Physically Active (KAPA) intervention and its implementation plan was guided by Bartholomew’s Intervention Mapping approach. The intervention mapping approach involved (1) performing a needs assessment and developing intervention objectives using previous literature; (2) identifying theory-based intervention strategies from a systematic review and the National Institute of Clinical Excellence guidelines; and (3) designing the KAPA intervention and its implementation plan with the guidance from an expert steering group.

**Results:**

The KAPA intervention comprised of six group sessions of motivational interviewing, delivered monthly by trained and mentor-supported falls prevention practitioners. Intervention sessions lasted up to 90 min and were delivered in community settings over a 6-month duration. Participant manuals, illustrated exercise books, physical activity diaries and pedometers supported the KAPA intervention.

**Conclusions:**

The intervention development process, consisting of Bartholomew’s Intervention Mapping approach and the input from an expert steering group, was successful in creating the evidence-based KAPA intervention ready to be evaluated in a feasibility trial.

**Supplementary Information:**

The online version contains supplementary material available at 10.1186/s40814-021-00844-w.

## Introduction

One third of adults aged over 65 years old, and half of adults aged over 80, fall each year and between 5 and 20% result in injury or hospitalisation [1–3]. In England, falls in older adults contribute to 255,000 emergency hospital visits yearly and an NHS annual spend of £2.3 billion in falls-related treatment [3–5]. Declines in physical activity results in cumulative muscle weakness, poor balance and physical impairments which markedly increase falls risk in older adults [6, 7]. To help improve health and physical function, the UK’s Chief Medical Officers recommend that older people perform 150 min of moderate to vigorous physical activity (MVPA) and two strength and balance exercise sessions weekly [8]. Physical, mental and social independence is retained when older adults autonomously remain active [9]. Yet, recent estimations show that 13% of men and 10% of women over 65 years old, and less than 5% of people over 75, meet the physical activity guidelines [10].

Falls prevention exercise programmes that target strength and balance exercise have been shown to reduce falling rates in older adults by 21% [11, 12]. However, longitudinal research findings suggest that older adults rarely maintain physical activity beyond 12 months after exercise programmes such as these end [13–16]. As a result, over time improvements in strength and balance are lost, and falls risk increases. The results of randomised controlled trials suggest that behaviour change interventions can help motivate older people to keep active after general physical activity promotion programmes end [17–29], but there is less certainty about whether physical activity can be maintained following specialised falls prevention exercise programmes [30–32].

The Falls Management Exercise (FaME) programme is a structured evidence-based exercise class that contains age appropriate exercises for older adults [16]. The ProAct 65+ trial found that an increased proportion of participants achieved 150 min of MVPA weekly in response to FaME [16]. Yet, the longitudinal findings showed that between 12 and 24 months after FaME classes came to an end the proportion of people meeting the MVPA target decreased, and 15.5% performed no minutes of MVPA weekly [16]. Additionally, research findings suggest that physical activity maintenance interventions may be ineffective in helping maintain physical activity increases after falls prevention exercise programmes end [30–33].

Currently, it is unknown what strategies would best maintain physical activity in older people exiting FaME programmes. This suggests that an intervention is needed to help educate, motivate and support older adults to remain physically active after FaME programmes end. The aim of this study was to develop an evidenced-based physical activity maintenance intervention. The intervention aimed to help older adults continue performing 150 min of MVPA, and two sessions of strength and balance exercise, per week after the completion of the FaME programme. The intervention developed was called Keeping Adults Physically Active (KAPA) and was delivered by 10 postural stability instructors to 50 FaME programme users attending 8 classes in Derby City, Leicestershire and Rutland. This paper describes the step-wise process of developing the evidence-based KAPA intervention.

## Methods

**Fig. 1:**
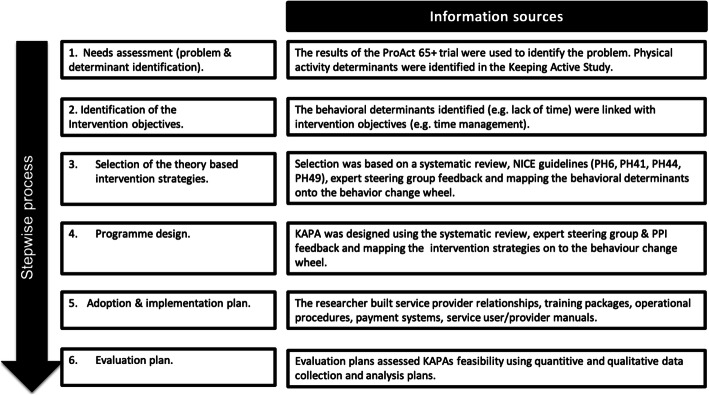
Development of the KAPA intervention using Bartholomew’s Intervention Mapping Approach. Based on the steps of Intervention Mapping Framework [35] Footnote**:** NICE—National Institute for Health and Care Excellence, PPI—People & Public Involvement

Bartholomew’s Intervention Mapping approach systematically integrates behaviour determinants, theory and research findings to develop interventions and their implementation and evaluation plans [34, 35]. The development of the KAPA intervention and its implementation plan was guided by the iterative six-step intervention mapping approach, as outlined in Fig. 1 [34, 35]. The findings of a literature review, previous qualitative research, government guidelines and an expert steering group supported the KAPA intervention development process [32].

### Step 1: needs assessment

Step 1 involved (i) identifying the problem and justifying the need for the KAPA intervention and (ii) identifying the determinants surrounding physical activity maintenance in falls prevention exercise programme completers. Justifying the interventions need was outlined in the introduction and will not be further discussed here. The determinants surrounding physical activity maintenance in FaME completers were identified in an ongoing study by the same research team (the Keeping Active study). The Keeping Active Study assessed both non-modifiable (i.e. demographic information) and modifiable (i.e. behavioral) determinants to physical activity maintenance in 30 older adults who had completed falls prevention exercise programmes, including the FaME programme [36, 37]. The study used semi-structured interviews to explore the barriers and facilitators for physical activity maintenance. The results suggested that factors surrounding motivation, self-efficacy autonomy, enjoyment, positive feedback, positive evaluation of physical activity (i.e. physical benefits and social interaction) and the development of habitual physical activity behaviours promoted physical activity maintenance [37]. The KAPA intervention was based solely on these modifiable determinants. These findings were used to help develop the KAPA intervention by extracting, tabulating and categorising the physical activity determinants into themes of physical, psychological, environmental and social factors. This formed the foundations of the matrix table on which the KAPA intervention was created (see Supplementary material 1, column 1 of the intervention matrix table).

### Step 2: identification of the intervention objectives

In step 2, the researchers correlated the physical activity determinants with potential solutions at participant level to support physical activity maintenance (i.e. performance objectives). For each physical activity determinant a performance objective was identified by asking the question “what do the participants need to do to affect this determinant?” [38]. Each performance objective was added onto the matrix. In order to identify the changes needed at provider level (i.e. change objectives) the question was asked “what do the service providers need to do to promote participant change?”. For example, developing new physical activity routines and habits was a physical activity determinant recognised in the Keeping Active Study [37]. The performance objective mapped to this determinant was for participants to “repeatedly perform new physical activity behaviours to help create new habits”. Whereas, the change objective was for service providers to “facilitate the development of behaviour repetition and relapse and prevention plans”. Performance and change objectives were mapped against each physical activity determinant on the matrix (Supplementary material 1, column 2 & 3 of the intervention matrix table). This step was important to ensure that actions to effect change were identified in order to achieve the desired intervention outcomes.

### Step 3: selection of theory-based intervention strategies

The goal of step three was to identify appropriate theory-based intervention strategies to achieve the intervention objectives. During this stage, a behaviour change theory and its associated intervention strategies were chosen to underpin the intervention. There is currently a lack of evidence supporting specific behaviour change theories for physical activity maintenance in older adults [39]. Hence, the behaviour change wheel was chosen as it incorporates multiple behaviour change theories [40, 41]. The behaviour change wheel categorises the opportunity, capability and motivation of specific populations to perform a given behaviour, otherwise known as the COM-B analysis. The COM-B analysis is centred at the core of the wheel and links to the intervention functions on the wheels next layer. The intervention functions most likely to evoke change are then selected. The intervention functions contain a mix of relevant behaviour change techniques (aka the behaviour change wheel’s intervention strategies) which are the “active components” that support individuals in making a behaviour change [40, 42].

The physical activity determinants identified in the Keeping Active Study were categorised into the COM-B. This was then used to identify the intervention functions and behaviour change techniques (BCTs) on the behaviour change wheel [41]. All BCTs identified during this process were added onto the matrix of behaviour determinants, performance and change objectives (see Supplementary material 1, column 4 of the intervention matrix table).

### Step 4: programme design

The fourth step involved specifying, planning and organising the KAPA intervention. This included specifying the BCTs, delivery modes, training package and resources. It was important that the KAPA intervention was evidence based and acceptable to both the service providers and participants. An expert steering group was formed consisting of seven local authority physical activity service managers, nine postural stability instructors and a sports development officer. To help improve the feasibility, acceptability and operationalisation of the KAPA intervention the group provided feedback and guidance during the intervention development process using an informal consensus approach.

Effective BCTs and delivery modes were identified in the literature base (i.e. KAPAs underpinning systematic review [33], a systematic review on behaviour maintenance [43] and the National Institute for Clinical Excellence (NICE) guidelines for physical activity and behaviour change (PH6, PH41, PH44, PH49) [44–47]. These were highlighted as potential BCTs on the matrix table. See Table 1 which shows the evidence based BCT’s chosen.

**Table 1:** Table of evidenced-based BCTs highlighted on the matrix table and included in the KAPA intervention

Source of evidence supporting KAPAs BCTs	Behaviour change techniques																														
	1.1 Goal setting (behaviours)	1.2 Problem solving	1.3 Goal setting	1.4 Action planning	1.5 Review behaviour goal(s)	1.6 Discrepancy (behaviour & goal)	1.7 Review outcome goal(s)	1.8 Behavioral contract	1.9 Commitment	2.2 Feedback on behaviour	2.3 Self-monitoring of behaviour	2.4 Self-monitoring outcome(s) of behaviour	2.5 Monitoring outcomes of behaviour	2.7 Feedback on behaviour outcomes	3.1 Social support (unspecified)	4.1 Instruction on how to perform a behaviour	5.1 Information about health consequences	5.3 Info –social/ environmental consequences	6.1 Demonstration of the behaviour	7.5 Remove aversive stimulus	8.2 Behaviour substitution	8.3 Habit formation	9.2 Pros and cons	9.3 Comparative imagining of future outcomes	10.4 Social reward	10.7 Self incentive	10.9 self - reward	11.2 Reduce negative emotions	11.3 Conserving mental resources	12.5 Adding objects to the environment	15.3 Focus on past successes
KAPAs supporting systematic review [33]	X	X	X	X	X		X	X	X	X	X				X	X	X	X	X			X								X	
NICE PH6, behaviour change general approaches [47]	X	X	X	X			X			X	X	X	X	X	X																
NICE PH41, PA: walking and cyclin g[46]	X		X								X	X	X	X	X							X								X	
NICE PH44, PA brief advice for adults in primary care [45]	X	X	X			X											X	X													
NICE PH49, behaviour change individual approaches [44]	X	X	X	X						X	X	X	X	X	X										X	X	X				
Kwasnicka et al. [43]					X	X				X	X	X	X	X						X	X		X	X	X	X	X	X	X		X

**X** indicates that the behaviour change technique (BCT) is present in the source of evidence

**Fig. 2:**
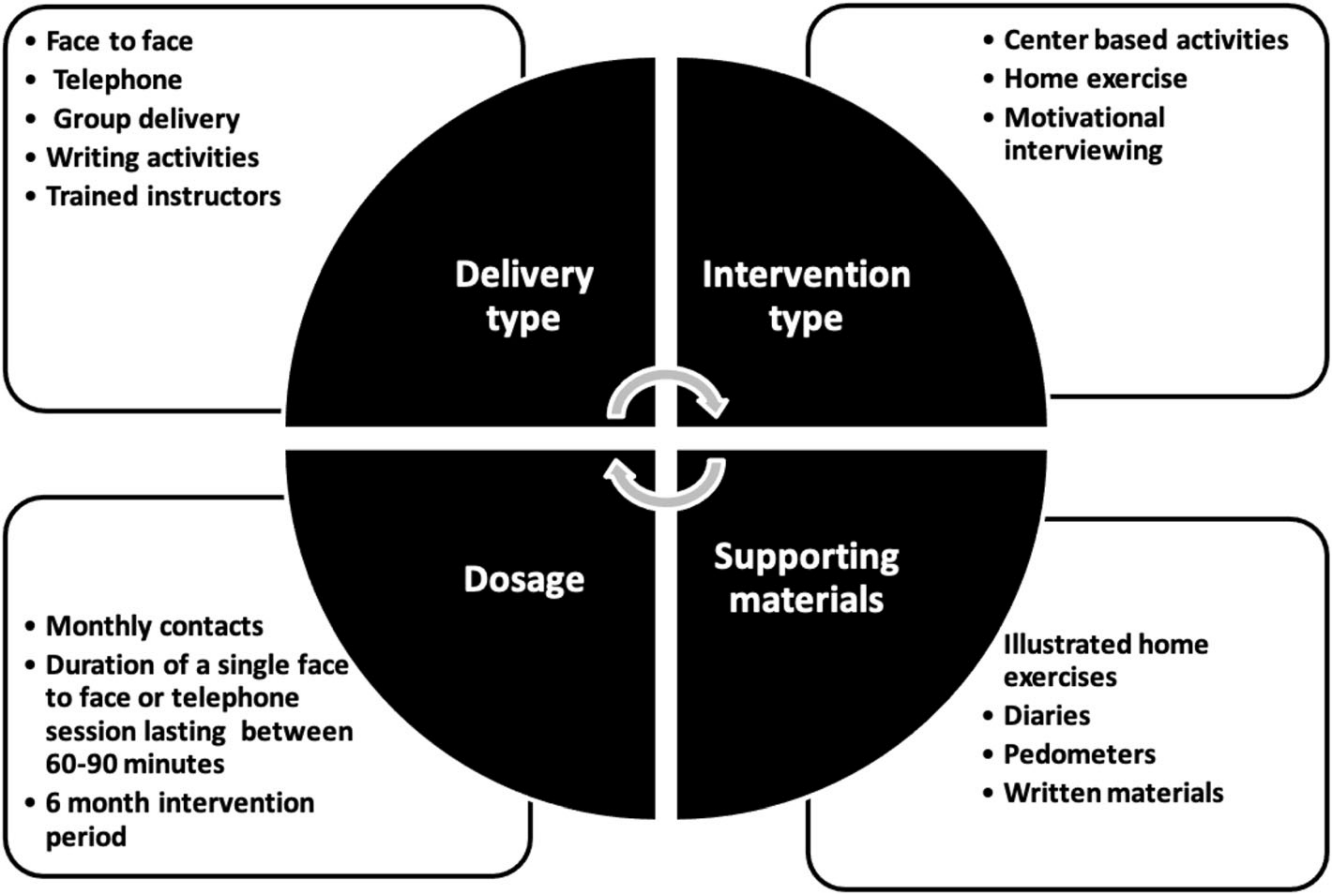
KAPA intervention framework developed from the systematic review and the NICE guidelines

An intervention framework, detailing potential BCTs and delivery methods, was developed using evidence from KAPA’s underpinning systematic review and NICE guidelines (Fig. 2) [5, 44, 45, 47]. In brief, the systematic review suggested that interventions were effective when delivered on a monthly or quarterly basis over a period of 6 months or more. Interventions delivered via multi-modal communication methods and a motivational interviewing approach were often effective. As were interventions supported by pedometers, exercise equipment and illustrative exercise sheets. NICE guidelines for physical activity also advocate the use of pedometers, monitoring and goal setting [45]. NICE guidelines for behaviour change recommend that staff should be adequately trained and mentors should support staff implementing interventions [44]. All information was depicted in the intervention framework and presented at two expert steering group meetings. Decisions surrounding the potential effectiveness, acceptability and the feasibility of operationalising each BCT and intervention delivery mode was agreed by the expert steering group and used to shape the final intervention (see Table 2).

**Table 2:** BCTs included in the KAPA intervention, in the order of delivery

Month Session content		BCT’s extracted from the intervention matrix
**1 Initial consultation: the aim of this session is to build a rapport with the participants, analyse their behaviours and develop effective action plans using motivational interviewing.**		
1.a Review health status		
1.b Explore physical activity knowledge and provide information on the physical activity guidelines		5.1
1.c Reflect on current physical activity levels (strength, MVPA, balance) and compare with physical activity guidelines, problem solve how to meet the guidelines		1.2, 1.6, 2.2
1.d Decisional balance analysis with mental imagery of two possible futures		9.2, 9.3
1.e Provide physical activity education and information about the physical activity services within the local community		5.3
1.f Introduce home exercise booklet and demonstrate exercises		4.1, 6.1
1.g Write a weekly physical activity plan (how, what, when and who with approach)		1.4
1.h Identify the barriers and facilitators relating to a person’s capability, opportunity and motivation to perform the plan		1.2
1.i Identify mechanisms of social support		3.1
1.j SMART goals for physical activity behaviours and outcomes		1.1, 1.3
1.k Modify goals and plans in accordance to self-reported commitment and confidence ratings		1.9
1.l Introduce the use of monitoring tools (i.e. physical activity diaries and pedometers)		2.3, 2.4, 12.5
1.m Summaries the session, gain a signature of commitment and confirm date of next session		1.8, 1.9
**2 Follow-up session 1: the first follow-up meeting aims to facilitate adherence to the physical activity plan and goals.**		
2.a Review health status		
2.b Review physical activity diaries, attainment of goals and revise/adapt physical activity plans and goals.		1.4, 1.5,1.7, 2.2, 2.5
2.c Use a motivational interviewing approach to help problem solve high-risk situations where physical activity has not been performed and write “if then” plans		1.2, 1.4, 3.1
2.d Encourage the use of diaries		2.3
2.e Reinforce social support mechanisms		3.1
2.f Summaries the session and confirm date of next session		
**3 Follow-up session 2: the aim of the second follow-up meeting is to facilitate physical activity adherence and introduce behaviour maintenance strategies.**		
Repeat steps 2a, 2b and 2c performed in the first follow-up meeting (i.e. session 2).		
3.a Relapse prevention strategies: (i) continued use of monitoring systems, (ii) reflecting on past successes, (iii) reflecting on lapses to identify high-risk situations and (iv) develop coping strategies		1.2, 1.4, 2.3, 2.4, 15.3
3.b Building new habits: (i) education of habit formation, (ii) self-review of poor physical activity habits, (iii) record habits in diary		2.3, 8.3
3.c Use of rewards for enacting good physical activity behaviours and goal attainment		10.7, 10.9
3.d Reflecting on enjoyment and satisfaction levels of being more active		11.2
Repeat steps 2d, 2e and 2f performed in the first follow-up meeting (i.e. session 2).		
**4 and 5 Follow-up sessions 3 and 4: the third and fourth follow-up meetings aim to help the participant build the self-regulation skills that they need to maintain the desired health behaviours.**		
Repeat steps 2a, 2b and 2c performed in the first follow-up meeting (i.e. session 2).		
4.a Problem solving and planning for possible future environmental and life changes		1.2, 1.4
4.b Reviewing habit diaries		2.2
4.c Problem solve and write plans to over-ride old habits (i.e. behaviour substitution plans and removing environmental triggers).		1.2, 1.4, 7.5, 8.2
5.a Identifying stress management strategies (e.g. rest breaks, Tia-Chi, mindfulness)		11.2
5.b Planning for times of low psychological resource levels (i.e. stress, grieving)		1.4, 11.2, 11.3
Repeat steps 2d, 2e and 2f performed in the first follow-up meeting (i.e. session 2).		
**6 Follow-up session 5: the last session aimed to build on the skills needed to maintain healthier physical activity habits**		
Repeat steps 2a, 2b and 2c performed in the first follow-up meeting (i.e. session 2).		
6.a Reflect on newly developed self-regulation skills		15.3
6.b Review physical activity plans and detail how people intend to maintain physical activity without further guidance		1.4
6.c Review achievements and provide praise		2.7, 10.4
6.d Close the session and sign a pledge of commitment		1.8, 1.9

### Step 5: adoption and implementation plan

Once the KAPA intervention was finalised, the next step was to develop an implementation plan. The intervention was operationalised by first developing the syllabus for the KAPA training programme. The training content related to the background knowledge and practical skills needed to deliver the KAPA intervention. The training resources included a trainee handbook, lecture slides, case studies and data collection forms. The training was delivered face to face over seven-hours in a group setting. A physiotherapist experienced in delivering physical activity programmes mentored individual postural stability instructors at their place of work. Mentoring sessions were provided at the postural stability instructors request and lasted between 30 and 45 min.

The standard operating procedure document was developed to outline the content and procedures of each of the six KAPA intervention sessions. Postural stability instructors were encouraged to refer back to the standard operating procedures before delivering each session to help improve fidelity. A participant manual was created and given to each participant to help guide them through the KAPA intervention. Many BCTs included in the KAPA intervention were similar to those delivered in England’s NHS Health Trainer service. Therefore, the NHS Health Trainer Handbook was used as a source of reference when developing the training syllabus, standard operating procedures and participant handbook [48].

### Step 6: generating an intervention evaluation plan

The final step was to generate an evaluation plan for the intervention. The feasibility of delivering the KAPA intervention was evaluated in a mixed-methods cluster randomised controlled feasibility study which evaluated recruitment, retention and attendance rates, self-reported physical activity and the feasibility and acceptability of the KAPA intervention. Details of the feasibility study methods and results are published elsewhere [49].

## Results

### KAPA intervention

Older adults exiting a 24-week FaME programme received six sessions of motivational interviewing over a 6-month period. Group sessions, aiming to provide social opportunities, lasted between 60 and 90 min and were delivered in accessible community-based venues. Participants unable to attend group sessions received the KAPA intervention via telephone.

KAPA participants were given a written resource containing information about locally available physical activity opportunities (e.g. age appropriate exercise classes, walking groups, racket sports, bowling), an illustrated postural stability exercise booklet (from Later Life Training) and information on lifestyle physical activity. Participants were given infographics explaining the UK’s Chief Medical Officers’ physical activity guidelines for adults. The guidelines define MVPA as being breathless, but still able to talk. The infographics depict different types of exercises that could result in MVPA (e.g. walking, cycling, swimming) and strength and balance exercise (e.g. resistance training, carrying shopping, bowls, yoga). Participants used the infographics to create a plan to meet the government recommendations of performing 150 min of MVPA and two sessions of strength and balance exercise weekly. Participants set physical activity goals and were encouraged to monitor their progress towards these by completing physical activity diaries and monitoring step count via pedometers.

The purpose of the second and subsequent sessions was to review physical activity diaries and provide feedback on goal attainment. Various BCTs were delivered throughout each of the six sessions to help support participants in continuing to be active (BCTs are outlined in Table 2). BCTs aimed to resolve ambivalence about remaining active, encourage adherence to the KAPA intervention and build the self-regulation skills needed to maintain physical activity habits. The precise detail of each of the six KAPA sessions can be viewed in the participant handbook provided in Supplementary material 2.

## Discussion

### Summary of the KAPAs intervention development process

The resultant intervention is a motivational interviewing programme aimed to help older adults remain physically active after the completion of the 24-week FaME programme. In line with the Chief Medical Officer’s guidance for physical activity, KAPAs primary goal was to support participants to achieve 150 min of moderate to vigorous physical activity (MVPA) and two sessions of strength and balance exercise per week [50]. We agree with the views of other intervention mapping users that the approach is useful in transparently building evidence-based interventions [51–56].

### Comparison with the previous literature

The results reported in KAPA systematic review suggest that physical activity maintenance interventions are ineffective in helping older adults exiting falls prevention exercise programmes to stay active [33]. Booster letters and review days delivered after an initial “hip fracture prevention” programme resulted in no significant differences in the proportion of participants who kept active for more than 30 min per week 2 years after the intervention ended [31]. Reductions in physical activity at 9-month follow-up were seen in another study that integrated unspecified relapse and prevention strategies into a fall’s prevention exercise programme [32]. Brief advice on remaining active after the completion of a falls programme did not result in physical activity levels being maintained at 9 or 12-months post-intervention [30].

KAPA differs from these previous interventions as it contains 6 sessions of postural stability instructor delivered motivational interviewing, supported by workbooks, goal setting, social opportunities and physical activity monitoring tools. Although no study has investigated these strategies for physical activity maintenance in falls-prevention exercise programme users, many have shown motivational interviewing [13, 18, 22, 24–26], physical activity monitoring tools, goal setting [13, 17–29, 57–60] and social support [28, 61–65] to help older adults remain active. This suggests that the KAPA intervention has the potential to help FaME programme users remain active. Certainly, the participants in the KAPA feasibility study reported that the peer support, exercise booklets and physical activity monitoring tools encouraged them to keep active [49]. However, a full-scale trial is needed to assess whether KAPA could significantly maintain physical activity increases after FaME programmes end.

### Strengths and limitations

Bartholomew’s Intervention Mapping approach is a systematic way of linking determinants, theory and intervention strategies to help develop a comprehensive evidence and theory-based intervention. The individual steps ensured that the development process was methodical, logical and transparent. Yet, a limitation of Bartholomew’s Intervention Mapping approach is that the application of each step is flexible, diverse and open to interpretation [52, 54, 55, 66–68]. Therefore, others may have gone through the same process and reached different conclusions on KAPAs intervention content.

In stage 1, it is common for researchers to collect qualitative information on behavioural determinants using service user focus groups and interviews [54, 55]. The data collected within the Keeping Active Study filled this void when developing the KAPA intervention as the physical activity determinants directly related to maintaining physical activity in older people exiting falls prevention exercise programmes. However, the needs of the participants in the ProAct 65+ trial maybe different to those taking part in local authority delivered FaME classes. Therefore, the physical activity determinants may have differed between the two participant groups and not all the determinants included may be relevant to the KAPA intervention.

During stage 2, the research team brainstormed a list of intervention objectives. It should be recognised that service users nor the expert steering group were included in this process. There are important distinctions between researcher, service user and provider perspectives. Thus, valuable service user and provider insights were likely missed which may have led to errors in identifying the most appropriate intervention objectives.

During stage 3, an intervention framework was created containing a simplified version of the evidence-based BCTs to help support the intervention development and refinement stage with the expert steering group. The expert steering group meetings were an integral part of KAPA’s development as the input from the expert steering group helped to ensure that the intervention was feasible and acceptable to deliver within each separate local authority. This was a particular strength as without the steering group’s input the intervention developed may not have been feasible to deliver. However, the steering groups involvement could have been strengthened by employing a nominal group approach, such as voting, over an informal consensus method, as this would have helped to quantify the level of agreement during the intervention development process [69].

During stage 4, the aim was to include BCTs to address each performance and change objective. It was outside the KAPA interventions scope to deliver BCTs for all the behaviour determinants identified. For example, it would have been difficult to address the socio-ecological behaviour mediators such as transport provision or to improve people’s memory. Addressing these determinants would require collaboration with many different types of services and professionals. Thus, KAPA’s intervention effectiveness maybe limited by not being able to provide resolutions for these determinants. Future interventions may benefit from including collaborators across multiple professions to help tackle issues that could limit intervention effectiveness.

### Implications for policy, practice and future research

The process of developing interventions in research and clinical practice has been criticised for its lack of conceptualisation and planning [70]. Failing to link behaviour determinants with appropriate theoretically based intervention strategies may in part explain the mixed outcomes of prior interventions [67]. Although Bartholomew’s Intervention Mapping approach is complex and resource and time intensive, researchers and practitioners would still benefit from using this transparent, responsive and systematic approach to help develop interventions with good scientific rigor [52, 70].

Reporting the intervention development process helps researchers, practitioners and policy makers to better understand the underpinning rationale, decision making processes and the methods used in developing interventions [71]. Publishing intervention development processes is integral to help to improve the transparency of the methodological rigor taken in developing interventions and can help practitioners and policy-makers assess whether pre-made interventions meet service-user needs [52, 71].

## Conclusion

Bartholomew’s Intervention Mapping approach provided a systematic framework to support the development of the KAPA intervention. The behaviour change wheel aided the mapping of evidenced-based BCTs onto the intervention matrix to help ensure that KAPA’s intervention components were appropriate to encourage older adults to remain active. The expert steering groups guidance was integral in shaping an intervention that was feasible for the postural stability instructors to deliver and acceptable for the FaME users to receive. The intervention development process was successful in developing an intervention ready to be evaluated in a feasibility trial. A full-scale randomised controlled trial is needed to assess whether the KAPA intervention is clinically effective in helping older adults remain active after the completion of FaME.

## Supplementary Information

**Additional file 1: Supplementary material 1**. Matrix of behaviour determinants, performance and change objectives and behaviour change techniques (BCTs).

**Additional file 2: Supplementary material 2**. Participant Handbook

## Data Availability

The datasets used (i.e. intervention matrix table and participant handbook) are included in the Supplementary materials 1 and 2.

## Abbreviations

BCTs: Behaviour Change Techniques
FaME: Falls Management Exercise
KAPA: Keeping Adults Physically Active
MVPA: Moderate to Vigorous Physical Activity
