# Supplementary material for: Keeping adults physically active after Falls Management Exercise (FaME) programmes end: development of a physical activity maintenance intervention
Source: Pilot Feasibility Stud. 2021 May 15;7:108. doi: 10.1186/s40814-021-00844-w (PMC8122574; doi:10.1186/s40814-021-00844-w)
Supplement: Supplementary file 1 — Additional file 1: Supplementary material 1. Matrix of behaviour determinants, performance and change objectives and behaviour change techniques (BCTs). [file 40814_2021_844_MOESM1_ESM.docx]

# Supplementary material 1. Matrix of behaviour determinants, performance and change objectives and behaviour change techniques (BCTs).

| **Behaviour determinants reported in Keeping Active Study** | **Performance objective (PO)** | **Change objective (CO)** | **Relevant BCTs identified in the literature and NICE guidelines (numerically coded in line with the v1 BCT taxonomy ^1^)** |
| --- | --- | --- | --- |
| **Physical capability determinants: Factors facilitating PA maintenance** | | | |
| Improved physical autonomy as a result of being physically active | PO1. A) Increase physical independence  B) Increase PA related self-efficacy. | CO1. A) Provide services to improve physical function and skill  B) Improve self-efficacy through PA practice | 1.4 Action planning  1.6 Discrepancy between current behaviour and goal  2.2 Feedback on behavior  2.3 Self-monitoring of behavior  2.4 Self-monitoring outcome(s) of behavior  4.1 Instruction on how to perform a behavior  6.1 Demonstration of the behavior  8.1 Behavioral practice/ rehearsal  8.3 Habit formation  8.7 Graded tasks  15.3 focus on past success |
| Positively evaluated the benefits of PA | PO2. A) Develop positive attitudes and beliefs about PA and its outcomes | CO2. A) Provide education on PA to help facilitate positive beliefs in PA outcomes | 1.2 Problem solving  1.4 Action planning  1.5 Review behavior goal(s)  1.7 Review outcome goal(s)  2.2 Feedback on behavior  2.3 Self-monitoring of behavior  2.4 Self-monitoring outcome(s) of behavior  3.1 Social support (unspecified)  8.1 Behavioral practice/ rehearsal  8.3 Habit formation  8.7 Graded tasks  11.2 Reduce negative emotions |
| Improved physical function as a result of being physically active | PO3. A) Improve physical function | CO3. A) Provide services to improve physical function and skill via PA practice | 1.1 Goal setting (behaviours)  1.2 Problem solving  1.3 Goal setting (outcome)  1.4 Action planning  1.5 Review behavior goal(s)  1.7 Review outcome goal(s)  2.2 Feedback on behavior  2.3 Self-monitoring of behavior  2.4 Self-monitoring outcome(s) of behavior  4.1 Instruction on how to perform a behavior  6.1 Demonstration of the behavior  8.1 Behavioral practice/ rehearsal  8.3 Habit formation  8.7 Graded tasks |
| Witnessing physical deterioration in friends and family members | PO4. A) Improve intention and motivation to be active  B) Change attitudes and beliefs | CO4. A) Increase recognition of the importance of PA  B) Use social comparison to highlight the importance of remaining PA | - 1. Goal setting (behaviours)   2. Problem solving   3. Goal setting (outcome)   1.4 Action planning  1.5 Review behavior goal(s)  1.7 Review outcome goal(s)  2.2 Feedback on behavior  2.3 Self-monitoring of behavior  2.4 Self-monitoring outcome(s) of behavior  3.1 Social support (unspecified)  8.3 Habit formation  9.2 Pros and cons  9.3 Comparative imaging of future outcomes |
| **Physical capability determinants: Factors acting as barriers to PA maintenance** | | | |
| Deterioration in physical health | PO5.  A) Increase knowledge of PA poor health  B) Increase self-efficacy to overcome barriers | CO5. A) Increase recognition of the importance of PA  B) Increase skills and confidence in overcoming barriers | 1.1 Goal setting (behaviours)  1.2 Problem solving  1.3 Goal setting (outcome)  1.4 Action planning  1.5 Review behavior goal(s)  1.7 Review outcome goal(s)  2.2 Feedback on behavior  2.3 Self-monitoring of behavior  2.4 Self-monitoring outcome(s) of behavior  3.1 Social support (unspecified)  4.1 Instruction on how to perform a behavior  6.1 Demonstration of the behavior  8.1 Behavioral practice/ rehearsal  8.3 Habit formation  8.7 Graded tasks |
| Fatigue | PO6. A) Increase self-efficacy to overcome barriers  B) Develop knowledge of PA and fatigue | CO6. A) Increase skills and confidence in overcoming barriers  B) Develop knowledge and pacing strategies | 1.1 Goal setting (behaviours)  1.2 Problem solving  1.3 Goal setting (outcome)  1.4 Action planning  1.5 Review behavior goal(s)  1.7 Review outcome goal(s)  2.2 Feedback on behavior  2.3 Self-monitoring of behavior  2.4 Self-monitoring outcome(s) of behavior  3.1 Social support (unspecified)  4.1 Instruction on how to perform a behavior  6.1 Demonstration of the behavior  8.1 Behavioral practice/ rehearsal  8.3 Habit formation  8.7 Graded tasks |
| **Psychological capability determinants: Factors facilitating PA maintenance** | | | |
| Motivation sourced by convenience of organised PA programmes | PO28. A) Increase motivation and intention to attend convenient PA opportunities  B) Make local PA programmes accessible | CO28. A) Increase intention to attend local PA programmes  B) Organise PA programmes that are considered convenient | 1.1 Goal setting (behaviours)  1.2 Problem solving  1.3 Goal setting (outcome)  1.4 Action planning  1.5 Review behavior goal(s)  1.7 Review outcome goal(s)  2.2 Feedback on behavior  2.3 Self-monitoring of behavior  2.4 Self-monitoring outcome(s) of behavior  3.1 Social support (unspecified)  4.1 Instruction on how to perform a behavior  5.1 Information about health consequences  5.3 Info –social/ environmental consequences  6.1 Demonstration of the behavior  8.1 Behavioral practice/ rehearsal  8.3 Habit formation  8.7 Graded tasks |
| Measurable activity with pedometers and tick charts | PO29. A) Monitor PA levels via monitoring tools  B) Increase knowledge on how to use technology | CO29. A) Increase intention to monitor PA  B) Education people on the use of technology and diaries | 1.1 Goal setting (behaviours)  1.3 Goal setting (outcome)  1.4 Action planning  1.5 Review behavior goal(s)  1.7 Review outcome goal(s)  2.2 Feedback on behavior  2.3 Self-monitoring of behavior  2.4 Self-monitoring outcome(s) of behavior  2.5 Monitoring outcomes of behaviour without feedback  2.7 Feedback on outcomes of behaviour  3.1 Social support (unspecified)  4.1 Instruction on how to perform a behavior  5.1 Information about health consequences  5.3 Info –social/ environmental consequences  6.1 Demonstration of the behavior  7.1 Prompts/cues  8.1 Behavioral practice/ rehearsal  8.3 Habit formation  8.7 Graded tasks |
| Increased self-efficacy | PO30. A) Increase PA related self-efficacy | CO30. A) Improve self-efficacy through PA practice | 1.1 Goal setting (behaviours)  1.2 Problem solving  1.3 Goal setting (outcome)  1.4 Action planning  1.5 Review behavior goal(s)  1.7 Review outcome goal(s)  2.2 Feedback on behavior  2.3 Self-monitoring of behavior  2.4 Self-monitoring outcome(s) of behavior  3.1 Social support (unspecified)  4.1 Instruction on how to perform a behavior  5.1 Information about health consequences  5.3 Info –social/ environmental consequences  6.1 Demonstration of the behavior  8.1 Behavioral practice/ rehearsal  8.3 Habit formation  8.7 Graded tasks |
| Development of a positive routine or habit | PO31. A) Manage habit formation | CO31. A) Facilitate behaviour repetition and develop relapse prevention strategies | 1.1 Goal setting (behaviours)  1.2 Problem solving  1.3 Goal setting (outcome)  1.4 Action planning  1.5 Review behavior goal(s)  1.7 Review outcome goal(s)  2.2 Feedback on behavior  2.3 Self-monitoring of behavior  2.4 Self-monitoring outcome(s) of behavior  3.1 Social support (unspecified)  4.1 Instruction on how to perform a behavior  5.1 Information about health consequences  5.3 Info –social/ environmental consequences  6.1 Demonstration of the behavior  7.1 Prompts/cues  7.5 Remove aversive stimulus  8.1 Behavioral practice/ rehearsal  8.2 Behaviour substitution  8.3 Habit formation |
| Improved confidence relating to a reduction in falls | PO32. A) Manage attitude towards in physical capacity  B) Manage confidence in physical function | CO32. A) Increase recognition of the importance of PA on physical function  B) Improve self-efficacy through PA practice | 1.1 Goal setting (behaviours)  1.2 Problem solving  1.3 Goal setting (outcome)  1.4 Action planning  1.5 Review behavior goal(s)  1.7 Review outcome goal(s)  2.2 Feedback on behavior  2.3 Self-monitoring of behavior  2.4 Self-monitoring outcome(s) of behavior  3.1 Social support (unspecified)  4.1 Instruction on how to perform a behavior  7.1 Prompts/cues  8.1 Behavioral practice/ rehearsal  8.3 Habit formation  8.7 Graded tasks |
| **Psychological capability determinants: Factors acting as barriers to PA maintenance** | | | |
| Poor beliefs towards own health and/or the ageing process | PO33. A) Develop a positive attitude about PA  B) Manage beliefs and expectations on the outcomes of poor health behaviours | CO33. A) Increase recognition of the importance of PA  B) Use social comparison to highlight the importance of remaining PA | 1.2 Problem solving  1.4 Action planning  1.5 Review behavior goal(s)  1.7 Review outcome goal(s)  2.2 Feedback on behavior  3.1 Social support (unspecified)  4.1 Instruction on how to perform a behavior  5.1 Information about health consequences  5.3 Info –social/ environmental consequences  8.1 Behavioral practice/ rehearsal |
| Others attitude towards ageing | PO34. A) Manage anticipated regret about others approval  B) Manage resistance to social pressure  C) Manage attitudes towards physical activity in later life years | CO34. A) Increase recognition of the importance of PA in older life years in social networks  B) Overcome barriers to social stigma  C) Reinforce health outcomes of physical inactivity | 1.2 Problem solving  1.4 Action planning  2.2 Feedback on behavior  3.1 Social support (unspecified)  5.1 Information about health consequences  5.3 Info –social/ environmental consequences |
| Depression/ low mood | PO35. A) Improve motivation to be active  B) Improve attitude towards being active | CO35. A) Educate on the use of reward systems  B) Educate on the positives of PA effects of mental state. | 1.1 Goal setting (behaviours)  1.2 Problem solving  1.3 Goal setting (outcome)  1.4 Action planning  1.5 Review behavior goal(s)  1.7 Review outcome goal(s)  2.2 Feedback on behavior  2.3 Self-monitoring of behavior  2.4 Self-monitoring outcome(s) of behavior  3.1 Social support (unspecified)  4.1 Instruction on how to perform a behavior  5.1 Information about health consequences  5.3 Info –social/ environmental consequences  7.1 Prompts/cues  8.1 Behavioral practice/ rehearsal  8.3 Habit formation  8.7 Graded tasks  10.4 Social reward  10.7 Self incentive  10.9 Self reward |
| Memory impairment | PO36. A) Manage coping responses  B) Develop habits | CO36. A) Develop cues/ stimulus and coping responses  B) Form automatic habits via repetition | 1.2 Problem solving  1.4 Action planning  2.2 Feedback on behavior  2.3 Self-monitoring of behavior  2.4 Self-monitoring outcome(s) of behavior  3.1 Social support (unspecified)  5.1 Information about health consequences  5.3 Info –social/ environmental consequences  7.1 Prompts/cues  8.1 Behavioral practice/ rehearsal  8.3 Habit formation |
| **Social Environment determinants: Factors facilitating PA maintenance** | | | |
| Social interaction | PO7. A) Improve social networks in PA classes  B) Increase intention to make friendships | CO7. A) Facilitate social support in PA services  B) Promote social interaction during classes | 1.2 Problem solving  1.4 Action planning)  3.1 Social support (unspecified)  6.1 Demonstration of the behavior  7.1 Prompts/cues  12.1 Restructuring physical environment  12.5 Adding objects to the environment |
| Enjoyment of community based structured exercise programmes | PO8. A) Manage provision of community-based classes  B) Deliver motivating services that improve skill and self-efficacy | CO8. A) Improve accessibility to community-based classes  B) Provide services to improve physical skill and self-efficacy | 1.1 Goal setting (behaviours)  1.2 Problem solving  1.3 Goal setting (outcome)  1.4 Action planning  1.5 Review behavior goal(s)  1.7 Review outcome goal(s)  3.1 Social support (unspecified)  6.1 Demonstration of the behavior  12.1 Restructuring physical environment  12.5 Adding objects to the environment |
| Motivation gained by exercising with others | PO9. A) Mobilise social support | CO9. A) Create mechanisms in services to reinforce social support | 1.1 Goal setting (behaviours)  1.2 Problem solving  1.3 Goal setting (outcome)  1.4 Action planning  1.5 Review behavior goal(s)  1.7 Review outcome goal(s)  2.3 Self-monitoring of behavior  2.4 Self-monitoring outcome(s) of behavior  3.1 Social support (unspecified)  6.1 Demonstration of the behavior  12.1 Restructuring physical environment |
| Partner’s motivating and encouraging one another | PO10. A) Mobilise social support  B) Manage and create social support networks  C) Reinforce accessing social support | CO10. A) Create mechanisms in services to reinforce social support networks  B) Create social support networks  C) Reinforce modes of social support outside of PA service venues | 1.1 Goal setting (behaviours)  1.2 Problem solving  1.3 Goal setting (outcome)  1.4 Action planning  1.5 Review behavior goal(s)  1.7 Review outcome goal(s)  2.3 Self-monitoring of behavior  2.4 Self-monitoring outcome(s) of behavior  3.1 Social support (unspecified)  6.1 Demonstration of the behavior  12.1 Restructuring physical environment |
| Positive feedback and evaluation of PA from friends and family | PO11. A) Mobilise social support  B) Manage and create social support networks  C) Manage access to social support networks  D) Manage feedback given by social support networks | CO11. A) Create mechanisms in services to reinforce social support networks  B) Create social support networks  C) Reinforce modes of social support outside of PA service venues  D) Improve knowledge of PA within social networks | 1.1 Goal setting (behaviours)  6.1 Demonstration of the behavior  1.3 Goal setting (outcome)  1.4 Action planning  1.5 Review behavior goal(s)  1.7 Review outcome goal(s)  2.3 Self-monitoring of behavior  2.4 Self-monitoring outcome(s) of behavior  3.1 Social support (unspecified)  12.1 Restructuring physical environment |
| **Social Environment determinants: Factors acting as barriers to PA maintenance** | | | |
| Caring roles prevented participation in structured organised PA. | PO12. A) Plan and manage coping responses  B) Manage intention to remain physically active | CO12. A) Create relapse prevention plans  B) Increase knowledge on different types of PA | 1.1 Goal setting (behaviours)  6.1 Demonstration of the behavior  1.2 Problem solving  1.3 Goal setting (outcome)  1.4 Action planning  1.5 Review behavior goal(s)  1.7 Review outcome goal(s)  2.3 Self-monitoring of behavior  2.4 Self-monitoring outcome(s) of behavior  3.1 Social support (unspecified)  7.1 Prompts/cues  7.3 Reduce prompts/cues  12.1 Restructuring physical environment  12.5 Adding objects to the environment |
| Lack of time and PA not a priority | PO13. A) Manage competing demands on time  B) Improve beliefs on the priority of PA and health | CO13. A) Acknowledge demands on time and demonstrate ability to time manage  B) Increase recognition of the importance of PA | 1.1 Goal setting (behaviours)  1.2 Problem solving  1.3 Goal setting (outcome)  1.4 Action planning  1.5 Review behavior goal(s)  1.7 Review outcome goal(s)  1.8 Behaviour contract  1.9 Commitment  2.3 Self-monitoring of behavior  2.4 Self-monitoring outcome(s) of behavior  3.1 Social support (unspecified)  7.1 Prompts/cues  7.3 Reduce prompts/cues  12.1 Restructuring physical environment  12.5 Adding objects to the environment |
| Social isolation | PO14. A) Mobilise social support  B) Manage and create social support networks within services  C) Manage access to social support networks | CO14. A) Create mechanisms in services to reinforce social support networks  B) Create social support networks within PA services  C) Reinforce access to social support within PA services. | 1.2 Problem solving  1.4 Action planning  3.1 Social support (unspecified)  6.1 Demonstration of the behavior  7.1 Prompts/cues  12.1 Restructuring physical environment  12.5 Adding objects to the environment |
| **Physical Environmental determinants: Factors facilitating PA maintenance** | | | |
| Location and availability of exercise programmes | PO15. A) Manage access to local PA programmes | CO15. A) Deliver PA programmes in local/accessible venues | 1.2 Problem solving  1.4 Action planning  3.1 Social support (unspecified)  12.1 Restructuring physical environment  12.5 Adding objects to the environment |
| Proximity to venues offering different types of exercise classes | PO16. A) Manage access to a variety of local PA programmes | CO16. A) Deliver a variety of PA programmes in local/accessible venues | 1.2 Problem solving  1.4 Action planning  3.1 Social support (unspecified)  12.1 Restructuring physical environment  12.5 Adding objects to the environment |
| Technology (apps, smart phones and pedometers) provided motivation and encouragement. | PO17. A) Monitor PA levels via technology  B) Increase knowledge on how to use technology | CO17. A) Increase intention to monitor PA  B) Provide education on technology | 1.1 Goal setting (behaviours)  1.2 Problem solving  1.3 Goal setting (outcome)  1.4 Action planning  1.5 Review behavior goal(s)  1.7 Review outcome goal(s)  2.2 Feedback on behavior  2.3 Self-monitoring of behavior  2.4 Self-monitoring outcome(s) of behavior  3.1 Social support (unspecified)  4.1 Instruction on how to perform a behavior  6.1 Demonstration of the behavior  8.1 Behavioral practice/ rehearsal  8.3 Habit formation  12.5 Adding objects to the environment |
| Having transport | PO18. A) Manage access to transport | CO18. A) Explore car sharing with people without transport | 1.2 Problem solving  1.4 Action planning  3.1 Social support (unspecified)  12.1 Restructuring physical environment  12.5 Adding objects to the environment |
| Cost or affordability | PO19. A) Manage access to affordance PA programmes | CO19. A) Improve access to affordable PA programmes | 1.2 Problem solving  1.4 Action planning  3.1 Social support (unspecified)  12.1 Restructuring physical environment  12.5 Adding objects to the environment |
| **Physical Environmental determinants: Factors acting as barriers to PA maintenance** | | | |
| Distractions in the home reduce people’s commitment towards home-based exercises | PO20. A) Manage competing demands in the home  B) Overcome barriers to increasing PA | CO20. A) Demonstrate the ability to manage competing demands in the home  B) Identify barriers and ways to overcome them. | 1.1 Goal setting (behaviours)  1.2 Problem solving  1.3 Goal setting (outcome)  1.4 Action planning  1.5 Review behavior goal(s)  1.7 Review outcome goal(s)  2.2 Feedback on behavior  2.3 Self-monitoring of behavior  2.4 Self-monitoring outcome(s) of behavior  3.1 Social support (unspecified)  4.1 Instruction on how to perform a behavior  6.1 Demonstration of the behavior  7.1 Prompts/cues  7.3 Reduce prompts/cues  8.1 Behavioral practice/ rehearsal  8.3 Habit formation  12.1 Restructuring physical environment  12.5 Adding objects to the environment |
| Lack of advertisement of local PA opportunities | PO21. A) Improve peoples knowledge of how to find out about local community services  B) Manage knowledge mobilisation of local PA services | CO21. A) Improve peoples skills in researching local community services  B) Improve advertising of local PA services | 1.2 Problem solving  1.4 Action planning  2.2 Feedback on behavior  3.1 Social support (unspecified)  4.1 Instruction on how to perform a behavior  6.1 Demonstration of the behavior  8.1 Behavioral practice/ rehearsal  12.1 Restructuring physical environment  12.5 Adding objects to the environment |
| Not having transport | PO22. A) Manage access to transport | CO22. A) Explore car-sharing and local transport services | 1.2 Problem solving  1.4 Action planning  3.1 Social support (unspecified)  12.1 Restructuring physical environment  12.5 Adding objects to the environment |
| Poor weather | PO23. A) Overcome barriers to increasing PA within the home | CO23. A) Identify barriers and ways to overcome them. | 1.1 Goal setting (behaviours)  1.2 Problem solving  1.3 Goal setting (outcome)  1.4 Action planning  1.5 Review behavior goal(s)  1.7 Review outcome goal(s)  2.2 Feedback on behavior  2.3 Self-monitoring of behavior  2.4 Self-monitoring outcome(s) of behavior  3.1 Social support (unspecified)  4.1 Instruction on how to perform a behavior  6.1 Demonstration of the behavior  8.1 Behavioral practice/ rehearsal  8.3 Habit formation  8.7 Graded tasks  12.1 Restructuring physical environment  12.5 Adding objects to the environment |
| Class times | PO24. A) Manage access to local PA programmes | CO24. A) Deliver day-time classes for older adults | 1.1 Goal setting (behaviours)  1.2 Problem solving  1.3 Goal setting (outcome)  1.4 Action planning  1.5 Review behavior goal(s)  1.7 Review outcome goal(s)  2.2 Feedback on behavior  2.3 Self-monitoring of behavior  2.4 Self-monitoring outcome(s) of behavior  3.1 Social support (unspecified)  4.1 Instruction on how to perform a behavior  6.1 Demonstration of the behavior  8.1 Behavioral practice/ rehearsal  8.3 Habit formation  8.7 Graded tasks  12.1 Restructuring physical environment  12.5 Adding objects to the environment |
| Personal safety when attending evening classes | PO25. A) Manage access to local PA programmes | CO25. A) Deliver day-time classes for older adults | 1.2 Problem solving  1.4 Action planning  3.1 Social support (unspecified)  12.1 Restructuring physical environment  12.5 Adding objects to the environment |
| Uneven terrain or hard surfaces caused joint pain | PO26. A) Overcome barriers to increasing PA | CO26. A) Identify barriers and ways to overcome them. | 1.1 Goal setting (behaviours)  1.2 Problem solving  1.3 Goal setting (outcome)  1.4 Action planning  1.5 Review behavior goal(s)  1.7 Review outcome goal(s)  2.2 Feedback on behavior  2.3 Self-monitoring of behavior  2.4 Self-monitoring outcome(s) of behavior  3.1 Social support (unspecified)  4.1 Instruction on how to perform a behavior  6.1 Demonstration of the behavior  8.1 Behavioral practice/ rehearsal  8.7 Graded tasks  12.1 Restructuring physical environment  12.5 Adding objects to the environment |
| Cost or affordability | PO27. A) Manage access to free or affordable PA programmes | CO27. A) Improve access to affordable PA programmes | 1.2 Problem solving  1.4 Action planning  3.1 Social support (unspecified)  12.1 Restructuring physical environment  12.5 Adding objects to the environment |
| **Reflective motivation: Factors facilitating PA maintenance** | | | |
| Positively evaluated the benefits of PA | PO2. A) Develop positive attitudes and beliefs about PA and its outcomes | CO2. A) Provide education on PA to help facilitate positive beliefs in PA outcomes | 1.2 Problem solving  1.4 Action planning  1.5 Review behavior goal(s)  1.7 Review outcome goal(s)  2.2 Feedback on behavior  2.3 Self-monitoring of behavior  2.4 Self-monitoring outcome(s) of behavior  3.1 Social support (unspecified)  8.1 Behavioral practice/ rehearsal  8.3 Habit formation  8.7 Graded tasks |
| Witnessing physical deterioration in friends and family members | PO4. A) Improve intention and motivation to be active  B) Change attitudes and beliefs | CO4. A) Increase recognition of the importance of PA  B) Use social comparison to highlight the importance of remaining PA | - 1. Goal setting (behaviours)   2. Problem solving   3. Goal setting (outcome)   1.4 Action planning  1.5 Review behavior goal(s)  1.7 Review outcome goal(s)  2.2 Feedback on behavior  2.3 Self-monitoring of behavior  2.4 Self-monitoring outcome(s) of behavior  3.1 Social support (unspecified)  8.3 Habit formation |
| Motivation gained by exercising with others | PO9. A) Mobilise social support | CO9. A) Create mechanisms in services to reinforce social support | 1.1 Goal setting (behaviours)  1.2 Problem solving  1.3 Goal setting (outcome)  1.4 Action planning  1.5 Review behavior goal(s)  1.7 Review outcome goal(s)  2.3 Self-monitoring of behavior  2.4 Self-monitoring outcome(s) of behavior  3.1 Social support (unspecified)  6.1 Demonstration of the behavior  12.1 Restructuring physical environment |
| **Reflective motivation: Factors acting as barriers to PA maintenance** | | | |
| Others attitude towards ageing | PO34. A) Manage anticipated regret about others approval  B) Manage resistance to social pressure  C) Manage attitudes towards physical activity in later life years | CO34. A) Increase recognition of the importance of PA in older life years in social networks  B) Overcome barriers to social stigma  C) Reinforce health outcomes of physical inactivity | 1.2 Problem solving  1.4 Action planning  2.2 Feedback on behavior  3.1 Social support (unspecified)  5.1 Information about health consequences  5.3 Info –social/ environmental consequences |
| Motivation sourced by convenience of organised PA programmes | PO28. A) Increase motivation and intention to attend convenient PA opportunities  B) Make local PA programmes accessible | CO28. A) Increase intention to attend local PA programmes  B) Organise PA programmes that are considered convenient | 1.1 Goal setting (behaviours)  1.2 Problem solving  1.3 Goal setting (outcome)  1.4 Action planning  1.5 Review behavior goal(s)  1.7 Review outcome goal(s)  2.2 Feedback on behavior  2.3 Self-monitoring of behavior  2.4 Self-monitoring outcome(s) of behavior  3.1 Social support (unspecified)  4.1 Instruction on how to perform a behavior  5.1 Information about health consequences  5.3 Info –social/ environmental consequences  6.1 Demonstration of the behavior  8.1 Behavioral practice/ rehearsal  8.3 Habit formation |
| Measurable activity with pedometers and tick charts | PO29. A) Monitor PA levels via monitoring tools  B) Increase knowledge on how to use technology | CO29. A) Increase intention to monitor PA  B) Education people on the use of technology and diaries | 1.1 Goal setting (behaviours)  1.3 Goal setting (outcome)  1.4 Action planning  1.5 Review behavior goal(s)  1.7 Review outcome goal(s)  2.2 Feedback on behavior  2.3 Self-monitoring of behavior  2.4 Self-monitoring outcome(s) of behavior  3.1 Social support (unspecified)  4.1 Instruction on how to perform a behavior  5.1 Information about health consequences  5.3 Info –social/ environmental consequences  6.1 Demonstration of the behavior  7.1 Prompts/cues  8.1 Behavioral practice/ rehearsal  8.3 Habit formation  8.7 Graded tasks |
| Development of a positive routine or habit | PO31. A) Manage habit formation | CO31. A) Facilitate behaviour repetition and develop relapse prevention strategies | 1.1 Goal setting (behaviours)  1.2 Problem solving  1.3 Goal setting (outcome)  1.4 Action planning  1.5 Review behavior goal(s)  1.7 Review outcome goal(s)  2.2 Feedback on behavior  2.3 Self-monitoring of behavior  2.4 Self-monitoring outcome(s) of behavior  3.1 Social support (unspecified)  4.1 Instruction on how to perform a behavior  5.1 Information about health consequences  5.3 Info –social/ environmental consequences  6.1 Demonstration of the behavior  7.1 Prompts/cues  8.1 Behavioral practice/ rehearsal  8.3 Habit formation |
| Partner’s motivating and encouraging one another | PO10. A) Mobilise social support  B) Manage and create social support networks  C) Reinforce accessing social support | CO10. A) Create mechanisms in services to reinforce social support networks  B) Create social support networks  C) Reinforce modes of social support outside of PA service venues | 1.1 Goal setting (behaviours)  1.2 Problem solving  1.3 Goal setting (outcome)  1.4 Action planning  1.5 Review behavior goal(s)  1.7 Review outcome goal(s)  2.3 Self-monitoring of behavior  2.4 Self-monitoring outcome(s) of behavior  3.1 Social support (unspecified)  6.1 Demonstration of the behavior  12.1 Restructuring physical environment |
| Enjoyment of community based structured exercise programmes | PO8. A) Manage provision of community based classes  B) Deliver motivating services that improve skill and self0efficacy | CO8. A) Improve accessibility to community based classes  B) Provide services to improve physical skill and self-efficacy | 1.1 Goal setting (behaviours)  1.2 Problem solving  1.3 Goal setting (outcome)  1.4 Action planning  1.5 Review behavior goal(s)  1.7 Review outcome goal(s)  3.1 Social support (unspecified)  6.1 Demonstration of the behavior  12.1 Restructuring physical environment  12.5 Adding objects to the environment |
| Positive feedback and evaluation of PA from friends and family | PO11. A) Mobilise social support  B) Manage and create social support networks  C) Manage access to social support networks  D) Manage feedback given by social support networks | CO11. A) Create mechanisms in services to reinforce social support networks  B) Create social support networks  C) Reinforce modes of social support outside of PA service venues  D) Improve knowledge of PA within social networks | 1.1 Goal setting (behaviours)  6.1 Demonstration of the behavior  1.3 Goal setting (outcome)  1.4 Action planning  1.5 Review behavior goal(s)  1.7 Review outcome goal(s)  2.3 Self-monitoring of behavior  2.4 Self-monitoring outcome(s) of behavior  3.1 Social support (unspecified)  12.1 Restructuring physical environment |
| **Automatic motivation: Factors acting as barriers to PA maintenance** | | | |
| Fatigue | PO6. A) Increase self-efficacy to overcome barriers  B) Develop knowledge of PA and fatigue | CO6. A) Increase skills and confidence in overcoming barriers  B) Develop knowledge and pacing strategies | 1.1 Goal setting (behaviours)  1.2 Problem solving  1.3 Goal setting (outcome)  1.4 Action planning  1.5 Review behavior goal(s)  1.7 Review outcome goal(s)  2.2 Feedback on behavior  2.3 Self-monitoring of behavior  2.4 Self-monitoring outcome(s) of behavior  3.1 Social support (unspecified)  4.1 Instruction on how to perform a behavior  6.1 Demonstration of the behavior  8.1 Behavioral practice/ rehearsal  8.3 Habit formation  8.7 Graded tasks |
| Depression/ low mood | PO35. A) Improve motivation to be active  B) Improve attitude towards being active | CO35. A) Educate on the use of reward systems  B) Educate on the positives of PA effects of mental state. | 1.1 Goal setting (behaviours)  1.2 Problem solving  1.3 Goal setting (outcome)  1.4 Action planning  1.5 Review behavior goal(s)  1.7 Review outcome goal(s)  2.2 Feedback on behavior  2.3 Self-monitoring of behavior  2.4 Self-monitoring outcome(s) of behavior  3.1 Social support (unspecified)  4.1 Instruction on how to perform a behavior  5.1 Information about health consequences  5.3 Info –social/ environmental consequences  7.1 Prompts/cues  8.1 Behavioral practice/ rehearsal  8.3 Habit formation  8.7 Graded tasks  11.3 Conserving mental resources |
| Lack of time and PA not a priority | PO13. A) Manage competing demands on time  B) Improve beliefs on the priority of PA and health | CO13. A) Acknowledge demands on time and demonstrate ability to time manage  B) Increase recognition of the importance of PA | 1.1 Goal setting (behaviours)  1.2 Problem solving  1.3 Goal setting (outcome)  1.4 Action planning  1.5 Review behavior goal(s)  1.7 Review outcome goal(s)  2.3 Self-monitoring of behavior  2.4 Self-monitoring outcome(s) of behavior  3.1 Social support (unspecified)  7.1 Prompts/cues  7.3 Reduce prompts/cues  12.1 Restructuring physical environment  12.5 Adding objects to the environment |
| Distractions in the home reduce people’s commitment towards home-based exercises | PO20. A) Manage competing demands in the home  B) Overcome barriers to increasing PA | CO20. A) Demonstrate the ability to manage competing demands in the home  B) Identify barriers and ways to overcome them. | 1.1 Goal setting (behaviours)  1.2 Problem solving  1.3 Goal setting (outcome)  1.4 Action planning  1.5 Review behavior goal(s)  1.7 Review outcome goal(s)  1.8 Behaviour contract  1.9 Commitment  2.2 Feedback on behavior  2.3 Self-monitoring of behavior  2.4 Self-monitoring outcome(s) of behavior  3.1 Social support (unspecified) |

1.

1. Abraham C, & Michie, S. A taxonomy of behavior change techniques used in interventions. *Health psychology* 2009; **27**(3): 379.
