# Supplementary material for: Keeping adults physically active after Falls Management Exercise (FaME) programmes end: development of a physical activity maintenance intervention
Source: Pilot Feasibility Stud. 2021 May 15;7:108. doi: 10.1186/s40814-021-00844-w (PMC8122574; doi:10.1186/s40814-021-00844-w)
Supplement: Supplementary file 2 — Additional file 2: Supplementary material 2. Participant Handbook [file 40814_2021_844_MOESM2_ESM.docx]

# Supplementary material 2. Participant Handbook

**Dear** *(participant name)*

Firstly, we would like to thank you for agreeing to take part in this study.

As you are aware, many people become less physically active as they get older which can lead to poorer strength and balance and an increased risk of disability and falls. You have been taking part in an exercise programme that helps to improve strength and balance and reduce falling risk. However, these beneficial effects reduce over time in people who do not keep physically active after exercise programmes have ended.

The “Keeping Adults Physically Active Programme” has been created to help encourage people to continue being physically active so that they do not lose the exercise benefits gained. This programme involves using physical activity planning, motivation boosting and problem-solving techniques to help you over-come any barriers that you may face towards being active.

This handbook includes worksheets for you to complete during the session. These worksheets will allow you to keep a written record of each meeting, your physical activity plan and any goals that you set. In addition to this you will be asked to keep a written diary detailing the physical activities that you perform and any barriers that you face between sessions.

Task 1. Please fill in your contact details. This information will be kept confidential.

Name:.......................................................................................... Postcode:..................................................................................... GP:...............................................................................................

Class location: ...............................................

Class Instructor: ...............................................

Please list your past medical history (illnesses and hospitalizations):

Please list the names of your medications:

Have you experienced any recent changes (over the last 6 months) in your health or the medication that you are taking? If so, please discuss these with your class instructor and list them in the space below.

Task 2. **What physical activity do you do now?**

Please complete the below table to show the physical activities that you have performed over the last seven days.

| **Day** | **What is the activity that you do?** | **Morning, afternoon or evening?** | **How long do you do this for?** |
| --- | --- | --- | --- |
| **Monday** |  |  |  |
| **Tuesday** |  |  |  |
| **Wednesday** |  |  |  |
| **Thursday** |  |  |  |
| **Friday** |  |  |  |
| **Saturday** |  |  |  |
| **Sunday** |  |  |  |

Chief Medical Officer Physical Activity Guidelines, Department of Health.

**Physical Activity Guidelines**


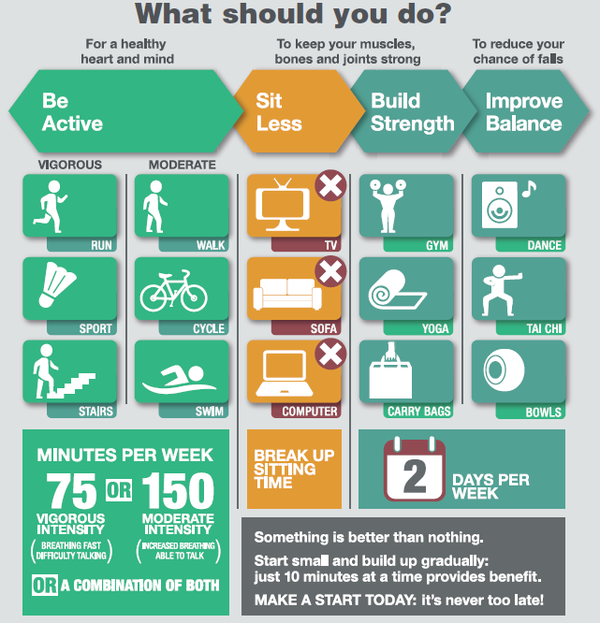


**What do the guidelines mean?**

Adults need to perform regular aerobic, strength and balance exercise to stay healthy or improve health. Adults aged 65 or older with no serious health conditions or mobility limitations should try to be active daily.

The physical activity guidelines advise that people should do:

**150 minutes** of **moderate activity** such as walking, cycling, swimming, aerobics or heavy housework each week. You should only count activities that get you **slightly out of breath** and that you did for at least **10 minutes at a time**.

***AND***

**Strength** and **balance** exercises on two or more days each week that will strengthen the legs, hips, torso and arms and improve your balance.

***AND***

It is also advised that you **sit less** and stand up and move about for at least one minute for every waking hour.

Task 3. Look back on the table which shows the physical activities that you have performed over the last seven days. Under the following headings list the activities that you did that involved:

**Moderate activity**.

**Strength activity.**

**Balance exercise.**

Do you think that you have done enough physical activity over the past 7 days to meet the physical activity guidelines? If not, what activities would you need to do more of and for how long?

| Type of activity you need to do | For how long? |
| --- | --- |
|  |  |

Task 4. **What are the benefits of keeping active?**

A way of helping people to decide whether to be physically active or not is to workout the benefits of being physically active. This task requires you to identify and write down the advantages and disadvantages of being physically active.

**List the disadvantages and advantages of being physically active.**

| **If I DO keep physically active:** | |
| --- | --- |
| Advantages | Disadvantages |
|  |  |

**List the advantages and disadvantages of NOT being physically active?**

| **If I DO NOT keep physically active:** | |
| --- | --- |
| Advantages | Disadvantages |
|  |  |

**How motivated are you to be physically active?**

On a scale of 1 to 10, how *motivated* are you to keep physically active?

1 = Not motivated at all, 10 = Very motivated. Circle a number on the line below:

**Not motivated at all** 1---2---3---4---5---6---7---8---9---10 **Very motivated**

**If you score less than 7, how certain are you that you want to remain less active?**

On a scale of 1 to 10, how *certain (sure)* are you that you want to remain less active? 1 = Not certain at all 10 = Very certain. Circle a number on the line below:

**Not certain at all** 1---2---3---4---5---6---7---8---9---**10 Very certain**

Task 5. **Physical activity planner.**

In the back of your pack you should find some suggestions of home-based and community based physical activities that you can perform. There are also exercise sheets that contain similar exercises to those that you have already been performing in your exercise class.

On the next page, complete the physical activity planner with the activities that you would like to perform each week. It may be a good idea to include the physical activities that you are currently performing as these already fit into your schedule. When choosing activities it may be worthwhile considering whether you will enjoy the activity, have transport to the venue and that you can make the class time.

To help you choose the correct activities we have colour coded the exercises as follows:

- GREEN: Moderate physical activities
- RED: Strength classes
- BLUE: Balance exercise

To meet the physical activity guidelines you would need to perform:

- 3 to 5 green activities weekly
- 2 red activities weekly
- 2 blue activities weekly

Physical Activity Planner.

| **Day** | **What types of activity will you do and how will you do it?** | **Where and what time?** | **How long will you do this activity for?** | **Who will you do this activity with?** |
| --- | --- | --- | --- | --- |
| **Monday** |  |  |  |  |
| **Tuesday** |  |  |  |  |
| **Wednesday** |  |  |  |  |
| **Thursday** |  |  |  |  |
| **Friday** |  |  |  |  |
| **Saturday** |  |  |  |  |
| **Sunday** |  |  |  |  |

Write down the activities that you are going to perform.

**How certain are you that you want to perform these activities?**  On a scale of 1 to 10, how *certain (sure)* are you that you want to perform these activities? 1 = Not certain at all 10 = Very certain. Circle a number on the line below

Not certain at all 1---2---3---4---5---6---7---8---9---10 Very certain

Task 6. Are there any barriers that may stop you from performing these activities?

a) **Physical barriers.** Is there anything about your home environment, the places that you visit, the things around you, your mobility or transport links or finances that may make it difficult for you to be active? Is there anything that you can do to change these barriers?

.....................................................................................................

.....................................................................................................

.....................................................................................................

b) **Unhelpful people and relationships.** Are there any people that you spend time or communicate with, people you care for or relationships that you have that may make it difficult for you to be active? What can you do to change this?

...............................................................................................................................................................................................................................................................................................................

c) **Unhelpful thoughts and feelings.** Are you having any thoughts or feelings that could make it difficult for you to be active? How can you change these thoughts and feelings?

..........................................................................................................................................................................................................

.....................................................................................................

**What things have made physical activity easier?**

a) **Helpful things in the environment.** Is there anything about your home environment, the places that you visit, the things around you or your transport links that has helped you to be active? How can you make the best use of these helpful things?

..........................................................................................................................................................................................................

.....................................................................................................

b) **Helpful people and relationships.** Have there been any people around you, people you spend time with or relationships that you have that have made it easier for you to be active? How can you make the best use of these helpful people?

..........................................................................................................................................................................................................

.....................................................................................................

c) **Helpful thoughts and feelings.** What thoughts or feelings have you had that have made it easier for you to be active? How can you make the best use of these helpful feelings?

..........................................................................................................................................................................................................

.....................................................................................................

Task 7. **Planning for difficult situations**

Do you foresee any situations that may make it difficult for you to be active? Are there any places, feelings, social situations or things in your environment that might tempt you to not be active?

For example: *“I get really tired on a Monday after I have babysat my grandchildren and this makes me not want to perform my home exercises.”*

Make a list of your difficult situations: .

.........................................................................................................................................................................................................

.....................................................................................................

This task requires you to make some plans about how you can lower the chance of these difficult situations interfering with your physical activity plan. To complete this task you will need to think of something that you could do to make each situation more manageable.

For example, **if** I babysit the grandchildren, **then** I will do my home exercises in the morning before I leave the house.

Make some “**if**-**then**” plans by filling in the table below. Listing each difficult situation and how you will cope with it.

| **Difficult situations**  **If...** | **Identify how you will cope with this situation**  **Then...** |
| --- | --- |
| *Example*  ***if*** *I babysit* | ***then*** *I will do my exercises before I go* |
|  |  |
|  |  |
|  |  |
|  |  |
|  |  |
|  |  |

Task 8. **My goals.**

**My long-term goal is:** ...............................................................................................................................................................................................................................................................................................................

**My daily/ weekly goals are:**

...............................................................................................................................................................................................................................................................................................................

**Is there anyone who can help you achieve your physical activity goals? How and when will you communicate with them?**

...............................................................................................................................................................................................................................................................................................................

**How confident are you in achieving this goal?**

On a scale of 1 to 10, how *confident are you in achieving this goal*?

1 = Not confident at all, 10 = Very confident. Circle a number on the line below:

**Not confident at all** 1---2---3---4---5---6---7---8---9---10 **Very confident**

Task 9. **Completing physical activity diaries.**

It is important for you to know how you are doing with your physical activity plan. Seeing your successes in your diary will help to motivate you to continue. On the other hand, if you are struggling to achieve your goal the diaries can be used to change your physical activity plans and goals to make them work for you.

There are two diaries that we would like you to complete. The first diary is for you to record the physical activities that you do throughout the week. This includes activities like walking, housework or gardening, going to the gym, exercise classes or doing exercise at home. You will also briefly record any barriers or difficulties that you have faced that have stopped you from being physically active. An example of a completed physical activity diary can be seen on page 18.

The second diary we would like you to complete is called an ABC Diary (**A**t first - **B**ehaviour- **C**onsequence Diary). In this diary you will record the barriers that you have faced in more detail. Your feelings and the people and environment around you can make it difficult for you to be physically active. Completing the ABC diary can help you to figure out what situations will trigger you to be physically active or not. This will help you to identify any situations that may cause you to have set backs. We can then help you to make plans to avoid these situations. An example of a completed ABC diary can be seen on page 19.

**An example of a completed physical activity diary**

| **Day** | **What activities did you do?** | **How long did you do this for?** | **Did you experience any difficulties towards being physically active? If so please complete the ABC form.** |
| --- | --- | --- | --- |
| **Monday** | *Walked to the shops* | *25 minutes* | *None* |
| **Tuesday** | *None* | *None* | *I was tired because I didn’t sleep well the night before.* |
| **Wednesday** | *I went to an exercise class* | *1 hour* | *none* |
| **Thursday** | *I did some gardening* | *45 minutes* | *none* |
| **Friday** | *I did home exercises* | *20 minutes* | *It was too cold to walk outside so I did home exercise instead.* |
| **Saturday** | *I went shopping with friend and took the stairs instead of the lifts.* | *2 hours* | *None- I felt good* |
| **Sunday** | *I walked to the shops* | *25 minutes* | *none* |

**Calculate your weekly activity:**

I spent 150 minutes doing moderate activity and I did 2 strength and balance sessions.

| **At first**  **What were you feeling, thinking, doing and who were you with?** | **What did you do?** | **What happened after?** |
| --- | --- | --- |
| I was feeling tired because I didn’t sleep well the night before. | I missed doing my home exercises | I enjoyed the rest but I felt guilty for not sticking to my physical activity plan. |
| I was shopping with my friend. We wanted to go to the second floor in the shop. She avoids using the stairs. I was tempted to use the lift but I remembered my goal and chose to take the stairs. | I walked up the stairs and met her on the second floor. | I was pleased that I did not feel tempted to take the lift. She congratulated me for being active. |

**An example of a completed ABC diary**

**What** will I record in my diary?

*Physical activity diary* .....................................................................................................

*ABC diary*

.....................................................................................................

**When** will I record it?

*Physical activity diary* .....................................................................................................

*ABC diary*

.....................................................................................................

**Where** will I keep my diaries? .....................................................................................................

Task 10. **My promise**

I promise that I will try to do the physical activity plan and meet my goals. I will bring my physical activity diaries to the next meeting.

Client signature: ............................................. Date..............................

**Physical Activity Instructor promise**

I promise that I will discuss with you your physical activity diary and how you got on when you bring it back at the next meeting.

Signature: ............................................. Date..............................

Date and time of next meeting: ……………………………………

**Tips for exercising at home.**

You do not have to spend money or go to a gym or exercise class to keep fit. The information below gives you some examples of physical activities that you can do at home.

**Moderate physical activity – GREEN activities**

Moderate physical activity should make you slightly breathless and/or sweaty. Your health is best improved when doing these activities for at least 10 minutes at a time.

- Use public transport or your car less, try **walking** or **cycling** instead.
- Use the **stairs** instead of the lifts and escalators.
- Do more **housework** or **gardening.**
- Try **dancing, tennis or walking football** as these are moderate physical activities that also help to improve your balance.

**Strength activities – RED activities**

- Doing **heavy gardening**, such as digging, will help to increase the strength in your legs.
- **Up-hill walking** or **up-hill cycling** helps to strengthen the muscles in your legs and will get your heart rate up so that you are also being moderately active.
- **Carrying shopping bags** helps to strengthen your arms.
- You can improve your arm and leg strength by practicing the **strength exercises** in your **home exercise booklet**.

**Balance activities – Blue activities**

- You can improve your balance by **playing bowls** or **dancing**.
- Continue to practice the **Tia Chi** exercises you did in your class.
- Doing the **balance exercises** in your **home exercise booklet** will help to improve your balance.

**Physical activity diary**

| Day | What activities did you do? | How long did you do this for? | Did you experience any difficulties towards being physically active? If so please complete the ABC form. |
| --- | --- | --- | --- |
| **Monday** |  |  |  |
| **Tuesday** |  |  |  |
| **Wednesday** |  |  |  |
| **Thursday** |  |  |  |
| **Friday** |  |  |  |
| **Saturday** |  |  |  |
| **Sunday** |  |  |  |

Fill in this diary everyday to record any physical activity that you do throughout the week. This includes activities like walking, housework or gardening, going to the gym, exercise classes or doing exercise at home. Only include activities that you spend 10 or more minutes performing. Write down how long you spent doing these activities and any barriers or difficulties that you have faced that has stopped you from being physically active.

**Calculate your weekly activity:**

I spent ………hours/minutes doing moderate activity and I did ………… strength and balance sessions.

**ABC Diary**

**A**t first (before) **B**ehaviour **C**onsequence

Your feelings and the people and environment around you can make it difficult for you to be physically active. Completing the ABC diary can help you to figure out what situations will trigger you to be physically active or not. Every time you perform a physical activity write down who you were with, how you felt and what you did next. Likewise fill in the diary every time you choose not to perform a physical activity that you had planned. This will help you to identify any situations that may cause you to have set backs. We can then help you to make plans to avoid these situations. The below table is an example of a completed diary.

| **At first**  **What were you feeling, thinking, doing and who were you with?** | **What did you do?** | **What happened after?** |
| --- | --- | --- |
| I was feeling tired because I didn’t sleep well the night before. | I missed doing my home exercises | I enjoyed the rest but I felt guilty that I did not stick to my physical activity plan. |

**ABC diary: A**t first –**B**ehaviour-**C**onsequence

| **At first**  **What were you feeling, thinking, doing and who were you with?** | **What did you do?** | **What happened after?** |
| --- | --- | --- |
|  |  |  |
|  |  |  |
|  |  |  |
|  |  |  |
|  |  |  |
|  |  |  |

**Date:**


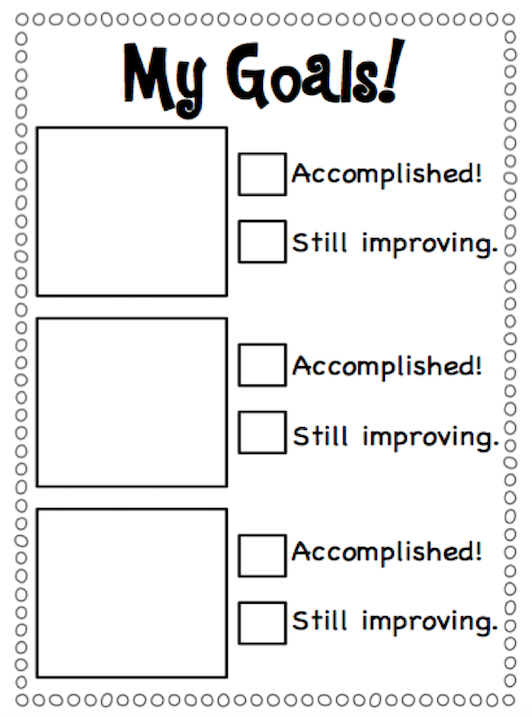


**Physical Activity Barriers.**

It is useful to identify the barriers that have prevented you from completing your physical activity plan. This worksheet will help you to problem solve the barriers that you have faced and develop plans to make them more manageable.

a) **Physical barriers.** Has there been anything about your home environment, the places that you visit, the things around you, your mobility or transport links or finances that have made it difficult for you to be active? What can you do to change these barriers?

.....................................................................................................

.....................................................................................................

.....................................................................................................

.....................................................................................................

b) **Unhelpful people and relationships.** Are there any people that you spend time or communicate with, people you care for or relationships that you have that has made it difficult for you to be active? What can you do to change this?

....................................................................................................................................................................................................................................................................................................................................................................................................................

c) **Unhelpful thoughts and feelings.** Are you having any thoughts or feelings that are making it difficult for you to be active? How can you change these feelings?

.....................................................................................................

.....................................................................................................

.....................................................................................................

**What things have made physical activity easier?**

a) **Helpful things in the environment.** s there anything about your home environment, the places that you visit, the things around you or your transport links that has helped you to be active? How can you make the best use of these helpful things?

.....................................................................................................

.....................................................................................................

.....................................................................................................

.....................................................................................................

b) **Helpful people and relationships.** Have there been any people around you, people you spend time with or relationships that you have that have made it easier for you to be active? How can you make the best use of these helpful people?

...............................................................................................................................................................................................................................................................................................................

c) **Helpful thoughts and feelings.** What thoughts or feelings have you had that have made it easier for you to be active? How can you make the best use of these helpful feelings?

....................................................................................................................................................................................................................................................................................................................................................................................................................

**Planning for difficult situations**

Have you found yourself in any situations that have made it difficult for you to be active? Have there been any places, feelings, social situations or things in your environment that have tempted you to not be active?

For example: *“I was really tired after I babysat my grandchildren and I felt too tired to perform my home exercises.”*

Make a list of your difficult situations: ..............................................................................................................................................................................................................................................................................................................................................................................................................................................................................................................................................................................................................................

This task requires you to make some plans on how you can lower the chance of these difficult situations interfering with your physical activity plan. To complete this task you will need to think of something that you could do to make each situation more manageable.

For example, **if** I babysit the grandchildren, **then** I will do my home exercises in the morning before I leave the house.

Make some “**if**-**then**” plans by filling in the table below. Listing each difficult situation and how you will cope with it.

| **Difficult situations**  **If...** | **Identify how you will cope with this situation**  **Then...** |
| --- | --- |
| *Example*  ***if*** *I babysit* | ***then*** *I will do my exercises before I go* |
|  |  |
|  |  |
|  |  |
|  |  |
|  |  |

**My New Goals**

Date:

**My new long-term goal is:** ...............................................................................................................................................................................................................................................................................................................

**My new daily/ weekly goals are:**

...............................................................................................................................................................................................................................................................................................................

**Is there anyone who can help you achieve your physical activity goals? How and when will you communicate with them?**

...............................................................................................................................................................................................................................................................................................................

**How confident are you in achieving this goal?**

On a scale of 1 to 10, how *confident are you in achieving this goal*?

1 = Not confident at all, 10 = Very confident. Circle a number on the line below:

**Not confident at all** 1---2---3---4---5---6---7---8---9---10 **Very confident**

New Physical Activity Plan.

Date:

Write down the activities that you are going to perform.

| **Day** | **What types of activity will you do and how will you do it?** | **Where and what time?** | **How long will you do this activity for?** | **Whom will you do this activity with?** |
| --- | --- | --- | --- | --- |
| **Monday** |  |  |  |  |
| **Tuesday** |  |  |  |  |
| **Wednesday** |  |  |  |  |
| **Thursday** |  |  |  |  |
| **Friday** |  |  |  |  |
| **Saturday** |  |  |  |  |
| **Sunday** |  |  |  |  |

**Planning to break bad habits (ABC Diary)**

Bad habits place your health at risk and prevent you from achieving your health goals. Habits are automatic behaviours that occur in response to familiar situations (for example, like getting in the car to go to the local shop, instead of walking). Habits are usually triggered by stress, boredom and tiredness.

Quite often people are unaware of their physical activity habits which makes it difficult to break bad habits. Complete an ABC habit diary over the next month to help identify the bad physical activity habits that you perform. Bring this diary with you in your next session so we can problem solve how to break these habits.

| **At first**  **What were you feeling, thinking, doing and who were you with?** | **What did you do?** | **What happened after?** |
| --- | --- | --- |
| I had a busy day and I was tired so I wanted to relax. | I watched TV for 4 hours without standing up. | My arthritic knee stiffened up and hurt when I stood up to go to bed. |
| I was agitated because my husband/wife drank the last of the milk and did not leave me any for my morning coffee. | I drove to the shop up the road to get the milk. | I enjoyed my cup of coffee and calmed down. |

*Example: ABC Habit Diary*

**ABC habit diary**

**Date:**

**A**t first (before) **B**ehaviour **C**onsequence

| **At first**  **What were you feeling, thinking, doing and who were you with?** | **What did you do?** | **What happened after?** |
| --- | --- | --- |
|  |  |  |
|  |  |  |
|  |  |  |
|  |  |  |
|  |  |  |

Rewards

Treating yourself when you have performed an activity or made progress towards achieving a goal can motivate you to continue being physically active. You can reward yourself when you have achieved any daily, weekly or long-term goals or any other smaller successes like remembering to complete your diary. Your rewards don’t have to cost money as these can be as simple as listening to your favourite music or reading a chapter of a book.

Try not to choose unhealthy rewards. For example, rewarding yourself with a chocolate biscuit every time you walk 10 minutes may not be good for your health if you walk for 10 minutes three times a day.

Use this table to list the rewards that you will give yourself for the activities that you perform and goals that you achieve.

| **The activities/ goals that I will reward myself for achieving** | **The rewards that I will give myself** |
| --- | --- |
|  |  |
|  |  |
|  |  |
|  |  |
|  |  |
|  |  |

Reflecting on your successes

Reminding yourself of personal successes and achievements helps you to recognise your progress and how you have changed for the better. In turn this will motivate you to continue with your physical activity plan. This task will help you to identify the benefits that you are gaining from being physically active and how this makes you feel. Reflect on your own successes by completing the table on the next page.

**Example, of a completed reflection table.**

| What was your success? | What did you do to achieve this success? | Why was it a good experience? | How did the success make you feel? |
| --- | --- | --- | --- |
| I can now walk without a walking stick. | I performed my strength exercises twice a week. | This was a good experience as I have walked with a stick for two years and didn’t think that I would be able to walk independently again. | This makes me feel more independent and confident with my mobility levels. |
| My blood pressure has lowered | I walked everyday for 30 minutes. | This was a good experience as my doctor praised me for lowering my blood pressure by being more active. | This made me feel proud of myself for sticking to my walking goal. |
| I achieved my step count goal everyday for a month. | I used my pedometer to track my step count and walked to the shops and back daily. | This was good because I knew that this was benefiting my health and helping me to achieve my long-term goal. | I felt refreshed and I was motivated to walk more. |

Reflect on your successes by filling in the below table.

| What was your success? | What did you do to achieve this success? | Why was it a good experience? | How did the success make you feel? |
| --- | --- | --- | --- |
|  |  |  |  |
|  |  |  |  |
|  |  |  |  |
|  |  |  |  |

**Planning for environmental and life changes**

A holiday break, change of weather, having to care for others or changes in your health may affect your opportunity to be physically active. Forecasting and planning for these situations can help you to maintain your physical activity levels during difficult times.

Make a list of any situations that could affect your activity levels: ........................................................................................................................................................................................................................................................................................................................................................................................................................................................................................................................................................................................................................................................................................................................................................................................................................................

Now make some plans on how you can lower the chance of these situations interfering with your physical activity plan. To complete this task you will need to think of something that you could do to make each situation more manageable.

For example, **if** I go on holiday, **then** I will walk along the beach daily.

Make some “**if**-**then**” plans by filling in the table below. Listing each change of situation and how you will cope with it.

| **Change of situation**  **If...** | **How I will avoid or cope with them Then...** |
| --- | --- |
| *Example…..*  ***If*** *I have to care for my husband/wife* | ***then*** *I will not go to my exercise class on a Monday and instead do exercise at home.* |
|  |  |
|  |  |
|  |  |
|  |  |

**Planning to break bad habits (substitution plans)**

Replacing a bad habit with a different behaviour can help people to become healthier. For example, replacing chocolate with fruit snacks helps people to eat healthier and cycling to work instead of driving allows people to be more active.

Use your ABC habit diary to reflect on the situations and feelings that you have that trigger your bad physical activity habits. With your class instructor identify what alternative behaviours you can do in response to these triggers. You may also want to think of ways to remove these triggers from your environment.

***Plan to break bad habits.***

| **Bad habit** | **Trigger** | **New behaviour response or removal of trigger** |
| --- | --- | --- |
| *Example:*  *Rather than go up and down the stairs multiple times a day, I will leave the things to go upstairs at the bottom of the staircase and take them up when I go to bed.* | *Example:*  *Sometimes I am busy and sometimes I am being lazy.* | *Example:*  *I will take the items upstairs straightaway to put them away.* |
|  |  |  |
|  |  |  |

**My promise**

I promise that I will try to continue to do my physical activity plan and goals. I understand that if I become less physically active then I may lose the positive health benefits that I have gained.

Client signature: ............................................. Date.............................

Instructor signature: ...................................... Date..............................

**Please note that much of the content of this handbook was adapted from the NHS Health Trainer Handbook.**
